# Multiplex imaging analysis of the tumor immune microenvironment for guiding precision immunotherapy

**DOI:** 10.3389/fimmu.2025.1617906

**Published:** 2025-07-11

**Authors:** Feng Liu, Guiqing Li, Yi Zheng, Yanxia Liu, Kang Liu

**Affiliations:** ^1^ Faculty of Medical Images, Shandong Second Medical University, Weifang, Shandong, China; ^2^ Department of Oncology, Afiliated Hospital of Shandong Second Medical University, Weifang, Shandong, China; ^3^ Shandong Provincial Key Medical and Health Discipline of Affiliated Hospital of Shandong Second Medical University in Oncology, Weifang, Shandong, China

**Keywords:** tumor immune microenvironment (TIME), multiplex imaging, spatial profiling, immune cell interactions, immunotherapy biomarkers

## Abstract

Cutting-edge multiplex imaging technologies have significantly advanced our understanding of the tumor immune microenvironment (TIME), delivering nanometer-scale spatial resolution that illuminates previously inaccessible cellular interactions and organizational patterns. This mini-review discusses the latest multiplex imaging methods, including Imaging Mass Cytometry (IMC), Multiplexed Ion Beam Imaging (MIBI), Cyclic Immunofluorescence (CycIF), and Digital Spatial Profiling (DSP), emphasizing their roles in identifying spatial immune signatures predictive of immunotherapy responses. Clinical applications across various cancers—such as NSCLC, melanoma, breast cancer, colorectal cancer, and hepatocellular carcinoma—highlight how spatially resolved immune profiles can enhance patient stratification and treatment personalization. Notably, increased CD8^+^ T cell density and spatial colocalization with tumor cells have been broadly correlated with improved immunotherapy response and survival across multiple cancer types. Despite current technical and analytical challenges, ongoing technological advancements and integration with emerging methods like spatial transcriptomics and super-resolution imaging promise broader clinical utility, ultimately improving patient outcomes in precision immunotherapy.

## 1 Introduction

The advent of cancer immunotherapy has fundamentally reshaped modern oncology, empowering clinicians to reprogram the body’s immune defenses into a precision weapon against malignant cells (1–3). Despite breakthroughs in immune checkpoint inhibitors and adoptive cell therapies, significant clinical challenges remain due to heterogeneous patient responses and resistance mechanisms (4–6). The efficacy of immunotherapies critically depends on the intricate spatial organization of the TIME, a highly complex ecosystem composed of tumor cells, immune cells (such as T cells, B cells, macrophages, dendritic cells, and neutrophils), stromal cells, cytokines, and extracellular matrix components. The spatial heterogeneity within the TIME significantly influences tumor progression, immune evasion, and therapeutic responsiveness (7, 8). Traditional immunotherapy biomarkers such as PD-L1 expression, tumor mutational burden, or immune infiltration scores have proven inadequate to fully capture the complexity and dynamic interactions occurring within the tumor microenvironment (9).

The emergence of sophisticated multiplex imaging platforms, including Imaging Mass Cytometry (IMC), Multiplexed Ion Beam Imaging (MIBI), and Cyclic Immunofluorescence (CycIF), have enabled comprehensive spatial mapping of dozens of biomarkers at single-cell resolution (10, 11). These innovative methodologies enable comprehensive, single-cell resolution mapping of cell phenotypes, functional states, and precise spatial relationships among immune and tumor cells within intact tissue sections. By leveraging multiplex imaging, researchers can now decode spatial signatures predictive of immunotherapy efficacy, such as immune cell clustering patterns, tumor-immune cell proximity, and localized immune cell activation or exhaustion zones (12–14). Consequently, the spatial immune signatures identified through multiplex imaging hold great promise for stratifying patients likely to respond favorably to immune checkpoint blockade or other immunotherapeutic approaches.

This mini-review aims to summarize current progress in applying multiplex imaging technologies to decipher the spatial architecture and functional dynamics of the TIME, with a specific focus on how these insights can guide precision immunotherapy. We discuss technical advancements, key spatial immune signatures, clinical implications, and future directions for integrating these powerful imaging tools into personalized cancer immunotherapy strategies. In contrast to previous reviews that focus primarily on imaging techniques or basic tumor biology (10, 15, 16), this mini-review uniquely emphasizes the translational relevance of spatial immune features derived from multiplex imaging. By integrating tumor-specific clinical examples, we highlight how these spatial biomarkers are being applied to predict immunotherapy response, stratify patients, and guide precision treatment strategies.

## 2 Multiplex imaging technologies for spatial analysis of TIME

Recent advancements in multiplex imaging technologies have transformed our ability to comprehensively analyze the spatial dynamics within the TIME (17). These cutting-edge methods, including IMC, MIBI, CycIF, and DSP, enable simultaneous visualization of numerous biomarkers at single-cell resolution, providing unprecedented insights into cellular interactions and functional states (18–20). By mapping complex spatial patterns such as immune cell distributions, activation zones, and tumor-immune interfaces, multiplex imaging significantly enhances our capacity to identify predictive spatial immune signatures, facilitating more precise patient stratification and personalized immunotherapy strategies (21, 22).

**Figure 1:**
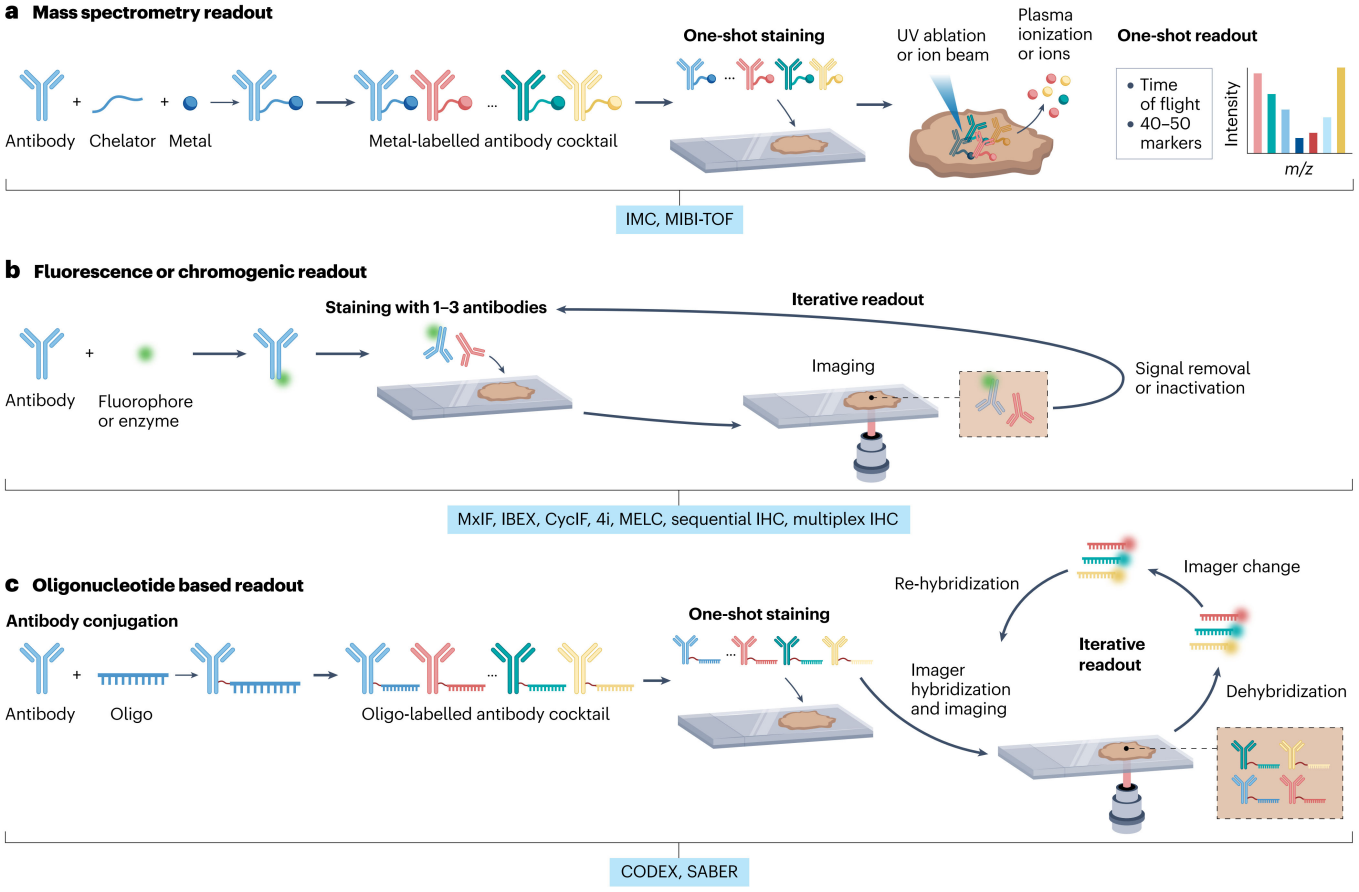
Schematic overview of multiplex imaging platforms used to analyze spatial protein expression in the TIME. **(a)** IMC and MIBI platforms utilize metal-conjugated antibodies detected by mass spectrometry, enabling highly multiplexed analyses with minimal spectral overlap. **(b)** Cyclic fluorescence-based imaging techniques such as CycIF and multiplex IHC rely on sequential antibody staining and stripping cycles, providing moderate multiplexing with broad accessibility. **(c)** Oligonucleotide-based imaging methods including CODEX and SABER use DNA-barcoded antibodies for high-plex fluorescence detection with spatial precision.

Mass spectrometry-based technologies, such as IMC and MIBI, utilize antibodies conjugated with metal isotopes (**Figure 1a**), which are detected by mass spectrometry to enable highly multiplexed analyses of up to approximately 40 markers (23, 24). These techniques offer superior specificity, minimal spectral overlap, and accurate quantification of marker expression, facilitating precise delineation of cell populations and states within intact tissues. This robust profiling capability supports in-depth exploration of immune cell interactions, activation states, and spatial distributions critical for understanding immune dynamics (25).

Cyclic fluorescence-based imaging methods, notably CycIF and multiplex immunohistochemistry (IHC), employ sequential cycles of antibody staining and imaging, as illustrated in **Figure 1b** (26, 27). These iterative processes enable analysis of up to 50 biomarkers while maintaining tissue morphology and structural integrity. CycIF and multiplex IHC are broadly applicable due to their integration into conventional fluorescence microscopy workflows, providing comprehensive spatial characterization of cellular neighborhoods, tissue architecture, and localized immune phenotypes (28).

Oligonucleotide-based imaging technologies, including CODEX and Signal Amplification by Exchange Reaction (SABER), use antibodies tagged with unique DNA oligonucleotide sequences (**Figure 1c**). Sequential hybridization with fluorescently labeled complementary probes permits detection of up to 60 markers (29, 30). CODEX and SABER offer exceptional multiplexing capabilities coupled with high spatial precision and excellent preservation of tissue structures, enabling detailed exploration of immune-tumor spatial relationships and functional interactions (31).

Digital Spatial Profiling (DSP) represents a specialized spatial analysis platform employing photocleavable oligonucleotide barcodes conjugated to antibodies or RNA probes. Targeted profiling of selected tissue regions is performed through controlled barcode cleavage and collection, enabling high-dimensional protein and RNA analysis without physical disruption of tissues (32). DSP excels in targeted, region-specific biomarker discovery, providing critical molecular context to spatially resolved immune landscapes. Spatial Molecular Imaging (SMI) utilizes iterative hybridization cycles of fluorescently barcoded oligonucleotide probes, enabling simultaneous high-dimensional detection of proteins or RNA transcripts at subcellular resolution within intact tissues (33). SMI significantly expands multiplexing capabilities and introduces transcriptomic profiling alongside protein analysis, offering deeper insights into the functional states and molecular mechanisms operative within spatially defined cellular environments. **Table 1** provides a comprehensive comparison of contemporary multiplex imaging platforms, delineating their respective capabilities, advantages, and constraints to guide researchers and clinicians in selecting optimal methodologies tailored to their specific investigative or diagnostic requirements.

**Table 1:** Comparison of multiplex imaging technologies for spatial analysis of the tumor immune microenvironment.

Technology	Resolution	Multiplex capability	Strengths	Limitations	Clinical translatability
Imaging Mass Cytometry (IMC)	~1 µm	Up to ~40 markers	High-dimensional data, minimal spectral overlap	Specialized instrumentation, costly reagents	Requires specialized facilities and is currently limited to research settings.
Multiplexed Ion Beam Imaging (MIBI)	~0.4 µm	Up to ~40 markers	Subcellular resolution, minimal spectral overlap	Complex data processing, specialized equipment	Requires highly specialized equipment, limiting routine clinical adoption.
Cyclic Immunofluorescence (CycIF)	~0.5-1 µm	30–50 markers	Broad accessibility, integrates into standard workflows	Potential tissue degradation over multiple cycles	Suitable for clinical labs due to compatibility with standard fluorescence microscopy.
CODEX (DNA-barcoded antibody imaging)	~0.5-1 µm	40–60 markers	Maintains tissue integrity, high multiplexing capacity	Complex optimization, extensive image processing	Increasing clinical adoption; requires trained personnel and controlled workflow integration.
Digital Spatial Profiling (DSP)	Region-specific	Dozens of markers	Targeted profiling, biomarker validation	Lacks single-cell resolution, requires prior ROI selection	Feasible in clinical settings with centralized testing models.
Spatial Molecular Imaging (SMI)	Subcellular	100+ proteins or RNAs	High-plex profiling; subcellular resolution; RNA/protein co-mapping	Long processing time; complex probe design; high cost	Promising but not yet suitable for routine clinical workflows.

Across the cited studies applying IMC, MIBI, CycIF, CODEX, DSP, and mIHC in various tumor types, several trends and limitations become apparent. IMC and MIBI offer high spatial resolution and deep multiplexing, and have been used to map immune-tumor interactions in melanoma and NSCLC. CycIF and CODEX are better suited for whole-slide imaging and large-area spatial profiling, though they can be labor-intensive or technically demanding. DSP, while lower in spatial resolution, provides scalable region-specific quantification and is gaining traction in prospective studies (34, 35). Multiplex IHC (mIHC) remains the most clinically accessible platform but is limited in multiplexing capacity and standardization. Despite methodological diversity, a common limitation lies in the lack of cross-platform comparability, standardized analysis pipelines, and prospective clinical validation. Together, these insights suggest the need for harmonization efforts and for embedding spatial immune biomarkers into clinical trial designs to enable robust patient stratification in precision immunotherapy (16).

Collectively, these multiplex imaging technologies profoundly enhance our understanding of the spatial complexities of TIME, providing critical insights into immune-tumor interactions that drive therapeutic efficacy. By systematically decoding these intricate spatial relationships, multiplex imaging significantly advances precision oncology, optimizing patient stratification and personalized immunotherapy strategies. A comprehensive comparison of these technologies is summarized in **Table 1**.

## 3 Decoding spatial immune signatures by multiplex imaging

Multiplex imaging technologies have transformed our capacity to dissect the spatial characteristics and functional intricacies of immune cells within tumor tissues. By utilizing high-dimensional spatial data, researchers have begun identifying unique spatial immune signatures that reflect complex cellular interactions and predict therapeutic outcomes.

Firstly, multiplex imaging allows precise characterization of immune cell subsets based on simultaneous detection of multiple markers, distinguishing activated, exhausted, regulatory, or effector immune phenotypes. For instance, CD8^+^ T-cells expressing high levels of granzyme B or interferon-γ within tumor tissues have been identified as markers for effective immune surveillance and favorable therapeutic responses (36). Conversely, elevated numbers of regulatory T cells (FoxP3^+^ Tregs) or exhausted T-cell populations expressing inhibitory receptors such as PD-1, TIM-3, and LAG-3 are frequently associated with poor prognosis and immunotherapy resistance.

A critical aspect deciphered by multiplex imaging is the spatial proximity between immune and tumor cells, which directly affects immunological interactions and clinical outcomes. These spatial arrangements are governed in part by underlying molecular cues such as chemokine gradients. For example, the CXCL9/CXCL10–CXCR3 axis has been shown to facilitate directional migration of effector CD8^+^ T cells toward tumor cores, promoting effective immune surveillance and enhancing responses to checkpoint blockade therapies (37, 38). Disruption or exclusion of such gradients may contribute to immune-desert or immune-excluded phenotypes, which are frequently associated with resistance to immunotherapy. Studies have illustrated that tumors characterized by tight spatial colocalization of tumor cells and cytotoxic T lymphocytes (CTLs) frequently correlate with improved clinical responses to checkpoint inhibitors (21, 39, 40). On the other hand, immune-exclusion phenotypes, characterized by T cells accumulating predominantly at the tumor margin and limited infiltration into tumor cores, typically predict resistance to immunotherapy.

Furthermore, multiplex imaging has facilitated the identification of distinct cellular neighborhoods or immune “hotspots”, clusters of interacting immune and stromal cells characterized by heightened immune activity. For instance, tertiary lymphoid structures (TLS)—organized clusters of B cells, dendritic cells, and T cells—detected through multiplex imaging have emerged as important predictive markers of immunotherapy response, indicating robust local immune activation and potential antitumor immune response centers (29).

Spatial immune signatures identified by multiplex imaging extend beyond individual immune subsets to patterns and configurations of cells in the tissue context. For example, distinct spatial patterns such as immune cell infiltration gradients, myeloid-enriched immune suppression zones, or tumor cell proliferation regions surrounded by immunosuppressive macrophages have shown robust predictive values. A recent multiplex imaging analysis in melanoma indicated that specific spatial immune patterns, notably the co-localization of CD8^+^ T-cells with antigen-presenting dendritic cells expressing CD11c, significantly correlate with favorable responses to anti-PD-1 therapy (39, 40).

In summary, by precisely decoding these spatial signatures within the tumor immune microenvironment, multiplex imaging technologies offer a nuanced understanding of immunological interactions and create new opportunities for predictive biomarker discovery, ultimately guiding more accurate patient stratification and personalized immunotherapy approaches.

## 4 Clinical insights from multiplex imaging in immunotherapy

Multiplex imaging technologies have significantly advanced our understanding of the TIME, enabling precise patient stratification and the development of tailored immunotherapy strategies. Clinical applications of multiplex imaging across various cancer types have underscored the significance of spatial immune profiling, thereby informing treatment choices and improving therapeutic outcomes.

### 4.1 Non-small cell lung cancer

Multiplex imaging studies in NSCLC have provided crucial insights into spatial immune characteristics predictive of immunotherapy responses (41). In a pivotal clinical study involving patients with advanced or metastatic NSCLC treated with PD-1 blockade combined with chemotherapy, multiplexed imaging revealed notable differences in immune cell composition and localization. Treatment responders exhibited markedly elevated infiltration densities of CD8^+^ cytotoxic T cells and CD68^+^ macrophages specifically within the tumor core region, revealing a distinctive spatial immune signature predictive of therapeutic success. These spatial immune signatures were strongly correlated with improved clinical outcomes, suggesting that immune cell infiltration into tumor cores plays a critical role in mediating therapeutic responses. Furthermore, this spatial information has practical implications for patient stratification, guiding clinicians in selecting patients most likely to benefit from combined immunotherapy and chemotherapy approaches. ​

### 4.2 Mismatch repair-deficient metastatic colorectal cancer

Multiplex imaging in patients with mismatch repair-deficient metastatic colorectal cancer undergoing anti-PD-1 therapy has highlighted significant correlations between spatial immune signatures and patient prognosis (29). Specifically, a higher density and closer proximity of CD8^+^ cytotoxic T cells to tumor cells have been associated with longer progression-free survival. This critical spatial arrangement underscores the importance of direct immune cell-tumor cell interactions in determining therapeutic efficacy, providing clinicians with powerful predictive biomarkers for identifying patients most likely to respond positively to immunotherapy. Such spatial immune profiling facilitates more informed treatment decisions, potentially improving clinical outcomes for this subset of colorectal cancer patients. ​

### 4.3 Breast cancer

Multiplex imaging has unveiled previously unrecognized complexity in the breast cancer immune landscape, establishing critical spatial relationships between immune cell distributions and therapeutic outcomes that were indiscernible with conventional techniques. Recent studies have demonstrated that specific spatial patterns, such as the formation of tertiary lymphoid structures (TLS) within tumors, are predictive of favorable responses to immunotherapies. Patients exhibiting these immune “hotspots”—rich in activated immune cells, including B cells, dendritic cells, and CD8^+^ T cells—typically experience better clinical outcomes and sustained therapeutic responses (42). Conversely, breast cancers characterized by immune-excluded phenotypes, where immune cells predominantly localize at tumor margins rather than infiltrating the tumor core, exhibit resistance to immunotherapy. These findings have significantly enhanced the ability to tailor treatment plans, guiding clinicians to select therapies or therapeutic combinations that could overcome resistance mechanisms associated with specific spatial immune patterns (43). ​

### 4.4 Hepatocellular carcinoma

Multiplex immunohistochemistry has been instrumental in dissecting the intricate spatial immune microenvironment of hepatocellular carcinoma (44). Detailed analyses have revealed distinct spatial distributions and interactions of immune cells, including regulatory T cells, cytotoxic T cells, and tumor-associated macrophages. These spatial relationships have critical implications for immunotherapeutic strategies, as they influence both tumor progression and treatment response. For example, regions enriched with suppressive immune cells correlate with poorer outcomes, suggesting potential therapeutic targets for combination therapies aimed at disrupting these immunosuppressive networks. These comprehensive insights have significant potential for optimizing personalized therapeutic approaches for HCC patients. ​

### 4.5 Melanoma

Multiplex imaging studies in melanoma have extensively demonstrated the clinical relevance of spatial immune cell arrangements in predicting responses to immune checkpoint inhibitors. A key finding is the favorable prognostic implication of CD8^+^ T cell clustering in close proximity to melanoma cells, strongly associated with positive outcomes following anti-PD-1 therapy. These insights have enabled clinicians to more accurately identify melanoma patients who are likely to benefit from specific immunotherapies. Moreover, multiplex imaging has identified immune exclusion patterns and exhausted immune cell populations that correlate with treatment resistance, offering potential avenues for developing combinatorial or alternative therapeutic strategies to overcome such resistance (39, 40). ​

Collectively, these detailed clinical examples across multiple cancer types highlight the transformative impact of multiplex imaging technologies in precision oncology. By elucidating the complex spatial dynamics of the TIME, multiplex imaging significantly enhances the predictive accuracy and efficacy of immunotherapy, paving the way for improved patient care and personalized cancer treatment strategies.

## 5 Current challenges and future perspectives in multiplex imaging for precision immunotherapy

Multiplex imaging has emerged as a transformative technology in tumor immunology, yet several technical and translational challenges continue to limit its routine clinical application. Technically, one major barrier is antibody validation. Multiplex assays require dozens of antibodies that must retain high specificity and compatibility within complex workflows. To address this issue, multicenter efforts such as the Human Protein Atlas project and the International Working Group for Antibody Validation (IWGAV) have proposed standardized validation strategies, including orthogonal methods, genetic knockdown models, and independent antibody cross-verification (11, 45). These initiatives aim to ensure reproducibility and reliability of antibody-based detection in high-plex imaging platforms. In fluorescence-based platforms like CycIF or CODEX, repeated staining cycles may degrade tissue integrity or reduce epitope availability, affecting data quality and reproducibility (46).

The formidable analytical challenges posed by these multidimensional datasets represent a substantial barrier to clinical implementation, requiring sophisticated computational pipelines to extract biologically and clinically meaningful insights. These technologies produce vast amounts of spatially resolved single-cell data, requiring advanced computational tools for segmentation, feature extraction, and interpretation. While machine learning and AI algorithms have improved analytical precision, they are not yet standardized or broadly accessible in clinical environments (14). Furthermore, the lack of unified pipelines and quality control measures hinders cross-institutional data comparability.

From a translational perspective, high equipment costs, specialized technical expertise, and long processing times limit implementation in routine pathology workflows. While the upfront costs for multiplex imaging are indeed substantial, centralized testing models—similar to those used in next-generation sequencing (NGS)—offer a scalable framework that can reduce per-sample costs over time. As adoption expands and workflows are streamlined, the economic burden may decrease, enabling broader clinical implementation and reimbursement justification in high-value oncology contexts. More importantly, regulatory frameworks for spatial biomarker interpretation are still underdeveloped (47). Without established clinical guidelines, it remains challenging to incorporate spatial immune signatures into decision-making processes for immunotherapy.

Notwithstanding these challenges, relentless technological innovation continues to push the boundaries of multiplex imaging, progressively overcoming barriers to clinical translation while revealing ever more sophisticated aspects of tumor-immune biology. Integration with spatial transcriptomics now enables simultaneous mapping of protein and gene expression, offering deeper insight into the tumor TIME (48). Additionally, super-resolution techniques like STORM and PALM can visualize nanoscale immune-tumor interactions, potentially identifying new therapeutic targets. Real-time and longitudinal imaging platforms such as intravital microscopy allow for dynamic tracking of immune responses over time.

Furthermore, the conceptual evolution of multiplex imaging in cancer immunology has moved from technical development and cell-type profiling toward clinically meaningful spatial biomarker discovery. Early studies emphasized spatial resolution and multiplexing capacity, while more recent work integrates spatial metrics—such as immune cell proximity, exclusion, and TLS formation—with therapeutic outcomes. The next phase involves embedding these markers into predictive models and trial designs. Emerging research is also increasingly focusing on integrating spatial proteomics with transcriptomics and AI-based image analysis. This progression reflects a paradigm shift from descriptive spatial biology to functional and predictive applications in precision oncology (11, 32, 49).

Moving forward, key priorities include the standardization of imaging protocols, development of clinically friendly analysis platforms, and validation of spatial immune signatures through prospective trials. Despite significant advancements, the ultimate goal of multiplex imaging analysis remains clinical translation. To achieve this, current research should prioritize validating spatial immune signatures in prospective clinical trials and integrating multiplex imaging into companion diagnostics. Efforts towards standardizing protocols for tissue preparation, image acquisition, analytical workflows, and biomarker interpretation are essential to ensure reproducibility across clinical centers. Moreover, multidisciplinary collaboration among pathologists, oncologists, imaging specialists, and bioinformaticians will accelerate the translation of multiplex imaging findings into practical tools that enhance patient stratification, optimize therapeutic choices, and ultimately improve clinical outcomes.

## 6 Conclusions

By providing an unprecedented window into the spatial organization of the TIME, multiplex imaging technologies have fundamentally altered our conceptual framework of tumor-immune-stromal interactions, revealing organizational principles with profound therapeutic implications. By accurately decoding complex spatial immune signatures, multiplex imaging has provided crucial biomarkers predictive of clinical responses to immunotherapies across multiple cancer types, such as melanoma, breast cancer, and lung cancer.

Despite existing challenges, including antibody standardization, assay reproducibility, analytical complexity, and limitations in clinical translation, the continued refinement of imaging platforms, computational algorithms, and integrative analysis methods promises to overcome these hurdles. Future advances involving combined approaches—such as integrating multiplex imaging with spatial transcriptomics, employing super-resolution microscopy, and utilizing real-time longitudinal imaging—will further enrich our biological understanding and enhance therapeutic precision. In aggregate, multiplex imaging stands at the forefront of a paradigm shift in precision oncology, offering transformative potential to revolutionize patient stratification strategies, optimize personalized immunotherapy approaches, and ultimately deliver improved survival outcomes across diverse cancer types.
